# Antimicrobial Effects of Non-Thermal Atmospheric Pressure Plasma on Oral Microcosm Biofilms

**DOI:** 10.3390/ijerph20032447

**Published:** 2023-01-30

**Authors:** Jiyeon Lee, Sungbo Cho, Hee-Eun Kim

**Affiliations:** 1 Department of Health Science, Gachon University Graduate School of Public Health, Incheon 21936, Republic of Korea; 2 Department of Electronic Engineering, Gachon University, Seongnam-si 13120, Republic of Korea; 3 Department of Dental Hygiene, Gachon University College of Health Science, Incheon 21936, Republic of Korea

**Keywords:** antimicrobial effect, atmospheric pressure plasma, biofilm, non-thermal plasma, oral disease, plasma

## Abstract

We comparatively evaluated the antibacterial effects of non-thermal atmospheric pressure plasma (NTAPP) on oral microcosm biofilms. Oral microcosm biofilms, which are derived from inoculation with human saliva, were cultured on 48 hydroxyapatite disks for 6 days. The prepared biofilms were divided into three different daily treatment groups: distilled water for 1 min, 0.12% chlorhexidine (CHX) for 1 min, and NTAPP for 5 min. Using a quantitative light-induced fluorescence-digital camera, the red fluorescence intensity of the biofilms was measured as red/green ratios (Ratio_R/G_) before and after treatment. Total and aciduric bacteria were counted as colony-forming units. Using live/dead bacterial staining, bacterial viability was calculated as the Ratio_G/G+R_. Ratio_R/G_ was approximately 0.91-fold lower in the NTAPP group than in the CHX group on day 1 of treatment (*p* = 0.001), and approximately 0.94-fold lower on both days 2 and 3 (*p* < 0.001). The number of total bacteria was higher in the NTAPP group than in the CHX group, but not significantly different. The number of aciduric bacteria was lowest in the CHX group (*p* < 0.001). However, bacterial viability was lowest in the NTAPP group. Restricted bacterial aggregation was observed in the NTAPP group. These findings suggest that NTAPP may more effectively reduce the pathogenicity of oral microcosm biofilms than 0.12% CHX.

## 1. Introduction

Biofilm-related oral diseases are initiated in inaccessible stagnation sites, where removing oral biofilms is challenging. Long-accumulated biofilms become thicker, and various physico-chemical gradients within the biofilms produce spatially heterogeneous communities [1] that can undergo microbiological shifts, which result in physiological changes as dynamic responses to environmental stress [2]. Such changes induce pathogenicity by interrupting the homeostasis within the biofilms [3]. Hence, several antimicrobial and chemotherapeutic agents have been utilized to inactivate older biofilms that cannot be removed solely through brushing of teeth.

However, older biofilms feature increased biomass, which can delay the dispersion of chemical agents. This is because the solute penetration rate in biofilms is inversely proportional to the density of the biomass [4]. Furthermore, solutes disperse more effectively in the outer layer of biofilms but have difficulty penetrating the inner layers [5]; therefore, older biofilms may contribute to the increasing resistance to antimicrobial agents. In addition, mouthwash with chemical agents disturbs the normal oral microflora, shifting to an acidic environment and promoting changes in oral microbial structure or reductions in community diversity [6,7]. Moreover, excessive use of such mouthwash may induce side effects such as vomiting and diarrhea. Therefore, an alternative to chemical agents is needed to control and treat localized sites showing the clinical signs of oral biofilms. For these reasons, we focused on non-thermal, atmospheric pressure plasma (NTAPP).

Plasma treatment, an advanced technology that has recently received increased attention in clinical practice, involves the application of non-thermal plasmas at approximately body temperature [8]. NTAPP consists of ionized gases, including charged and neutral species and electric fields [9,10]. Medically-relevant NTAPP sources and devices are usually dielectric barrier discharges and non-thermal, atmospheric pressure plasma jets [8]. In particular, because NTAPP operates on atmospheric pressure and the generated heat does not exceed 50 °C, the affected tissue can be directly treated without causing cell injury [9]. In dentistry, the applications of NTAPP include bonding to dentin and ceramics, curing of composites, tooth bleaching, surface activation of dental implants, antimicrobial therapeutic interventions, endodontology, and periodontology [11]. The antimicrobial effect of NTAPP is mainly attributed to the generation of reactive oxygen species (ROS) and reactive nitrogen species (RNS), including free radicals [9,12]. ROS and RNS target various structures of bacteria; for example, they etch the cell wall or disrupt the cell membrane with high electrostatic forces. Moreover, they facilitate the oxygenation of molecules such as lipids, proteins, polysaccharides, and nucleic acids in bacteria [13,14,15,16]. Recently, a hand-held device called a Plasma Pipette^®^ (Femto Science, Hwaseong, Republic of Korea), which can be used chairside, was introduced [17]. We focused on the potential use of NTAPP to locally target a specific site to control pathogenic biofilms.

Several recent studies have substantiated the antimicrobial effect of NTAPP using mono-species and multi-species biofilm models [12,18,19]. Blumhagen et al. [20] reported that applying argon-based NTAPP for 60 s to *Streptococci* or *Lactobacilli* biofilms led to complete sterilization. Koban et al. [21] reported that treating multi-species biofilms with an argon-based NTAPP jet for up to 600 s significantly reduced colony-forming units (CFUs). Furthermore, they stated that the antibacterial effect increased in a time-dependent manner. However, because more than 1000 species of bacteria form a microcosm in the oral cavity, the formation of biofilms by some species of bacteria lacks clinical relevance [22]. Hence, the antimicrobial effects of NTAPP should be more accurately assessed using an oral microcosm biofilm model that resembles the oral cavity instead of biofilms formed by particular bacterial species; however, such research is extremely rare.

The oral microcosm biofilm model is derived from human saliva and is representative of the interactions among metabolically heterogeneous microbial communities within the oral cavity [23]. For this reason, this model is recognized as an in vitro model that can reproduce the actual oral cavity biofilm [24]. Therefore, we aimed to enhance the clinical validity of the in vitro results by assessing the antimicrobial effects of NTAPP using an oral microcosm biofilm model. More specifically, we comparatively evaluated the antimicrobial effects of NTAPP against oral microcosm biofilms. Our null hypothesis was that there would be no significant difference in the antimicrobial effect of NTAPP on oral microcosm biofilms compared to that of chlorhexidine solution (CHX).

## 2. Materials and Methods

### 2.1. Sample Size Determination and Specimen Preparation

For the one-way analysis of variance (one-way ANOVA) of the three groups, the sample size was determined using the G*Power software version 3.1 (Heinrich-Heine-University Düsseldorf, Düsseldorf, Germany). Based on the results of a preliminary experiment, the sample size was calculated to be 48 for an effect size (f) of 0.47, probability of alpha error of 0.05, and power of 0.80.

A total of 48 hydroxyapatite disks (HA disk, Himed, NY, USA) with a diameter of 7 mm and a height of 2 mm were prepared. All HA disks were ground sequentially using 400–1200-grit sandpaper and a polishing machine (M-PREP 5TM, Allied High Tech Products, Inc., Compton, CA, USA). With reference to the literature [25], the HA disk was placed within the acrylic mold, and 1 mm of space was secured above the disk for biofilm formation. All disk specimens were then sterilized with ethylene oxide gas.

### 2.2. Formation of the Oral Microcosm Biofilm

**Figure 1:**
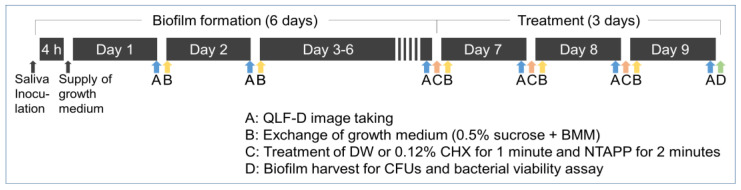
Formation of oral microcosm biofilm and treatment procedures (BMM, basal medium mucin; CFUs, colony forming units; CHX, chlorhexidine; DW, distilled water; NTAPP, non-thermal atmospheric pressure plasma; QLF-D, quantitative light-induced fluorescence-digital).

This study was approved by the institutional review board (IRB) of Gachon University (IRB No.1044396-202107-HR-165-01). To collect saliva, three healthy women without active caries or periodontal disease who had not used antibiotics in the past three months were recruited. The participants did not perform any form of oral hygiene for 24 h prior to the saliva collection, and collected stimulatory saliva by chewing on paraffin wax for 5 min. The collected saliva was filtered through glass wool (Duksan Chemicals, Ansan, Republic of Korea). Then, the HA disk specimens were placed on the first row of 24-well cell culture plates (SPL Life Sciences, Pocheon, Republic of Korea), and the disks were inoculated with 1.5 mL of filtered saliva. The saliva-inoculated, 24-well plates were incubated at 37 °C and 10% CO_2_ (BB15 CO_2_ incubator, Thermo Scientific, Waltham, MA, USA) for 4 h. Then, saliva on each specimen was carefully removed, after which fresh medium containing 0.5% sucrose (0.1 mL) and basal medium mucin (BMM, 1.4 mL) was added to each specimen. The 0.5% sucrose was prepared using saccharose (Daejung Chemicals & Metals Co., Ltd., Siheung, Republic of Korea). The BMM medium consisted of 2.5 g/L porcine mucin (Type III; Sigma Chemicals, MO, USA), 10.0 g/L proteose peptone (KisanBio, Seoul, Republic of Korea), 5.0 g/L trypticase peptone (KisanBio, Seoul, Republic of Korea), 5.0 g/L yeast extract (KisanBio, Seoul, Republic of Korea), 2.5 g/L potassium chloride (KisanBio, Seoul, Republic of Korea), 1 mmol/L urea (Georgiachem, GA, USA), 1 mmol/L arginine (Georgiachem, GA, USA), and 1 mg/L menadione (Sigma Chemicals, MO, USA). The final pH of the medium was adjusted to 7.0. This growth medium was replaced at the same time every day for 6 d; all well plates were stored at 37 °C with 10% CO_2_ (Figure 1).

### 2.3. Plasma Treatment

**Figure 2:**
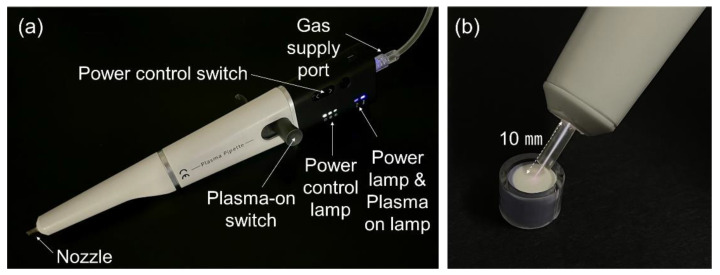
Plasma Pipette^®^. (**a**) The parts of the Plasma Pipette^®^. (**b**) Length of the white dotted line (10 mm) indicates the distance between the nozzle of the plasma flame and oral microcosm biofilm.

NTAPP was applied for 5 min on the specimens with biofilms using an argon gas-based Plasma Pipette^®^ (Figure 2a). The Plasma Pipette^®^ was operated under 4 W power, 10 kV voltage, and 100 kHz frequency, and the output pressure was set to 0.02 MPa. While applying NTAPP, the distance of plasma application was consistently maintained at 10 mm (Figure 2b). Biofilms not exposed to NTAPP were used as controls. The negative control group was treated with 1.5 mL of distilled water (DW) for 1 min, and the positive control group was treated with 1.5 mL of 1.2% CHX (Hexamedine, Bukwang Pharm. Co., Ltd., Ansan, Republic of Korea) for 1 min. After treatment, the antimicrobial substances were removed by washing all specimens with 1.5 mL of phosphate-buffered saline (PBS, Welgene, Gyengsan, Republic of Korea) three times. After all treatments, 1.5 mL of fresh growth medium was added. The treatment was applied once a day for 3 d, and the biofilm specimens were stored at 37 °C with 10% CO_2_ at all other times (Figure 1).

### 2.4. Analysis of Red Fluorescence Intensity

**Figure 3:**
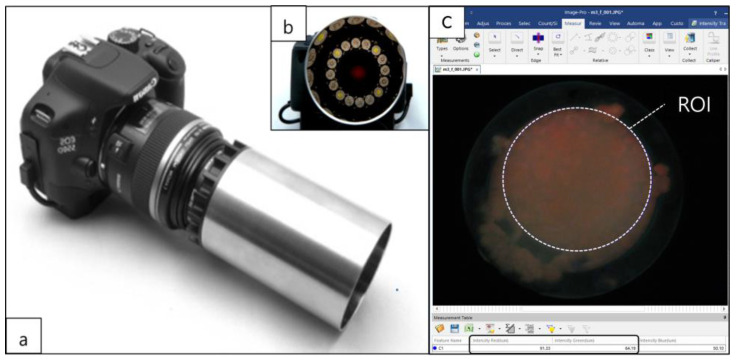
(**a**) Quantitative light-induced fluorescence-digital (QLF-D) camera. (**b**) Light sources of the QLF-D camera. (**c**) Region of interest (ROI) on fluorescence image of oral microcosm biofilm for analyzing red and green intensities.

To assess the intensity of red fluorescence in the biofilm, a fluorescence image was taken using the blue light of a quantitative light-induced fluorescence-digital (QLF-D Biluminator™2+, Inspektor Research Systems BV, Amsterdam, Netherlands) camera every day before changing the growth medium (Figure 3a,b). The settings for the blue light photograph were as follows: shutter speed, 1/60 s; aperture value, 7.1; and ISO speed, 1600. The distance between the lens and biofilm was maintained at 10 cm, and the photograph was taken in a dark room at 24 °C. The intensity of red fluorescence in the acquired images was analyzed using Image-PRO^®^ version 10 software (Media Cybernetics, Inc., Silver Spring, MD, USA). An identical region of interest was set for each biofilm image for analyzing red and green intensities (Figure 3c). The ratio of red to green intensities (Ratio_R/G_) was calculated, with a lower value indicating stronger antimicrobial activity.

### 2.5. Evaluation of Colony Count

At the end of the treatment period, the biofilm-covered HA disk specimens were rinsed with 1.5 mL PBS three times to remove any floating bacteria. Then, all specimens were transferred to conical tubes containing 1 mL of PBS. The tubes were vortexed (VM-96A; Lab Companion, Seoul, Republic of Korea) for 1 min, followed by ultrasound vibration (SHB-1025; Saehan Sonic, Seoul, Republic of Korea) for 1 min to prepare the biofilm supernatants. The supernatants were diluted in series (10^−1^–10^−6^) and were plated (100 µL) onto 5% brain heart infusion (BHI) agar (Becton Dickinson & Co., Franklin Lakes, NJ, USA) plates (final pH 4.8) and tryptic soy blood (TSB) agar (Becton Dickinson & Co., Franklin Lake, NJ, USA) plates (final pH 7.0) containing 5% sheep blood. After 72 h, the number of total and aciduric bacterial colonies were counted.

### 2.6. Bacterial Viability Assay

Bacterial viability was assessed using the LIVE/DEAD BacLight Bacterial Viability kit (LIVE/DEAD kit; Invitrogen^TM^, Waltham, MA, USA). As per the manufacturer’s instructions, SYTO-9 and propidium iodide (PI) was mixed in a 1:1 ratio and 3 µL of mixed stain solution was added to 1 mL of biofilm supernatant. The mixture of supernatant was incubated in a dark room at 37 °C for 15 min. Then, 5 µL of supernatant was dripped onto a slide glass and fixed with a cover slip. After setting the excitation light at 488 nm and 555 nm using ZEN 3.7 microscopy software (Carl Zeiss, Inc., Oberkochen, Germany), the images of the stained samples were taken with a confocal laser scanning microscope (CLSM; Zeiss LSM 700, Carl Zeiss, Inc.) at 100× magnification. To calculate bacterial viability, the green and red intensities of the entire images were analyzed using Image-Pro^®^. The ratio of the green-stained areas to the total area (Ratio_G/G+R_) was calculated to indicate bacterial viability; a higher value indicated higher viability.

### 2.7. Statistical Analysis

The data were analyzed using SPSS for Windows, version 26.0 (IBM Co., Armonk, NY, USA), and statistical significance was set at *p* < 0.05. Normality was tested for all outcomes. The interaction effects for each group according to the treatment agent and duration were analyzed using repeated-measures ANOVA, and differences between groups at the same treatment duration were analyzed using Tukey’s post-hoc test. The differences in total bacterial count and aciduric bacterial count according to the treatment agent were analyzed using one-way ANOVA, and the differences between the groups were analyzed using the Tukey’s and Games–Howell post-hoc tests.

## 3. Results

### 3.1. Red Fluorescence Intensity of Oral Microcosm Biofilms

**Figure 4:**
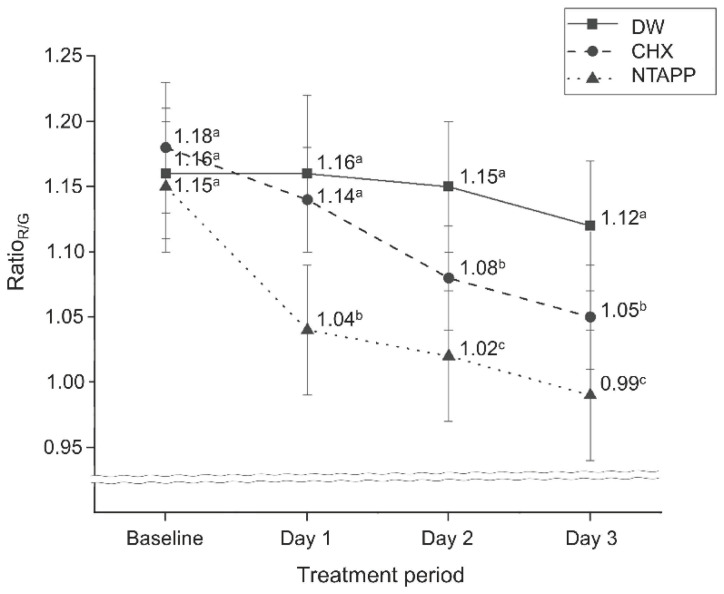
Differences in Ratio_R/G_ depending on treatment methods and periods on oral microcosm biofilms. Data are presented as means. ^a,b,c^ Different letters within the same column indicate significant differences between groups obtained using Tukey’s post-hoc analysis at α = 0.05. (DW, distilled water; CHX, chlorhexidine; NTAPP, non-thermal atmospheric plasma; Ratio_R/G_, the ratio of red to green pixels in fluorescence images captured by QLF-D).

**Figure 5:**
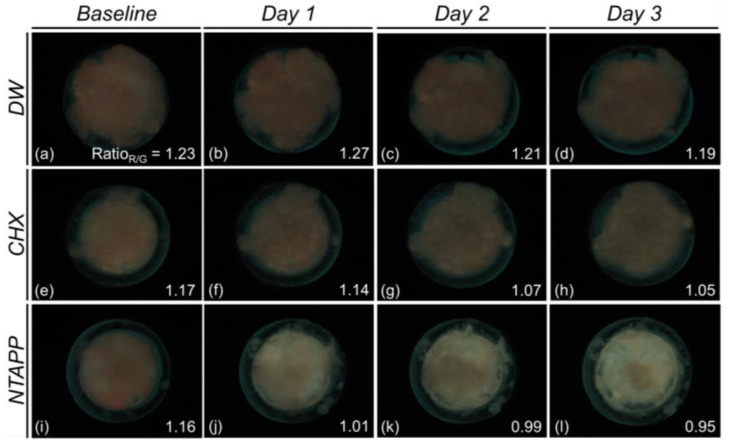
Representative fluorescence images of oral microcosm biofilms by QLF-D camera. The number in the lower right means Ratio_R/G_. (**a**–**d**) After DW treatment, (**e**–**h**), After CHX treatment, (**i**–**l**), After NTAPPJ treatment, (**a**,**e**,**i**), on baseline, (**b**,**f**,**j**), on treatment day 1, (**c**,**g**,**k**), on treatment day 2, (**d**,**h**,**l**), on treatment day 3. (CHX, chlorhexidine; DW, distilled water; NTAPP, non-thermal atmospheric pressure plasma; QLF-D, quantitative light-induced fluorescence-digital).

Regarding the interaction between the treatment agent and duration on the Ratio_R/G_ of biofilm, treatment duration influenced the antimicrobial effect of a treatment agent (*p* < 0.001). Furthermore, the NTAPP group had the lowest Ratio_R/G_ at all treatment durations (*p* < 0.001, Figure 4 and Figure 5). The Ratio_R/G_ on treatment day 1 was approximately 0.91-fold lower in the NTAPP group than in the CHX group (*p* = 0.001), showing that NTAPP had the highest antimicrobial effect. On both days 2 and 3, the Ratio_R/G_ was approximately 0.94-fold lower in the NTAPP group than in the CHX group, respectively (*p* < 0.001, Figure 4).

### 3.2. Total Bacterial and Aciduric Bacterial Counts

**Figure 6:**
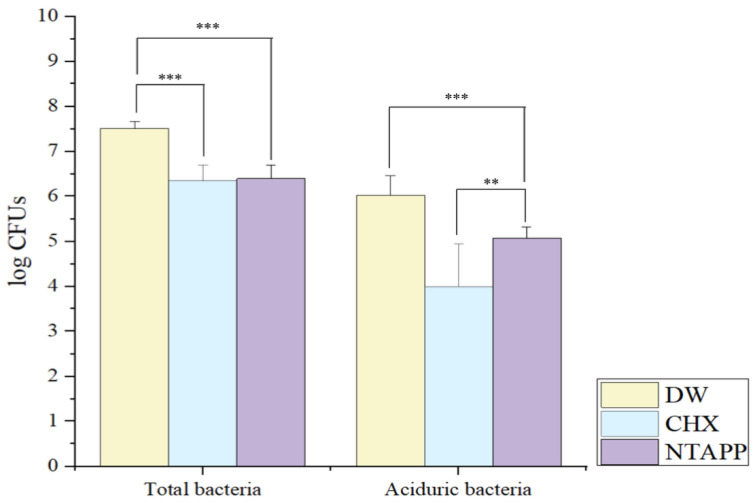
Colony-forming units of total and aciduric bacteria according to the treatment **: *p* < 0.01, ***: *p* < 0.0001 (CFUs, colony-forming units; CHX, chlorhexidine; DW, distilled water; NTAPP, non-thermal atmospheric pressure plasma).

Total bacterial and aciduric bacterial counts differed according to the treatment agent (*p* < 0.001, Figure 6). Total bacterial count differed between the NTAPP (6.39 ± 0.30) and CHX groups (6.36 ± 0.34) by 0.03 ± 0.45, but the difference was not statistically significant (*p* = 0.463). However, the total bacterial count was lower in the NTAPP group than in the DW group (7.51 ± 0.16) by 1.12 ± 0.34 (*p* < 0.001). The aciduric bacterial count differed between the NTAPP (5.07 ± 0.25) and CHX groups (4.00 ± 0.94) by 1.07 ± 0.97 (*p* = 0.001) and between the NTAPP and DW groups (6.03 ± 0.43) by 0.96 ± 0.58 (*p* < 0.001, Figure 6).

### 3.3. Bacterial Viability

**Figure 7:**
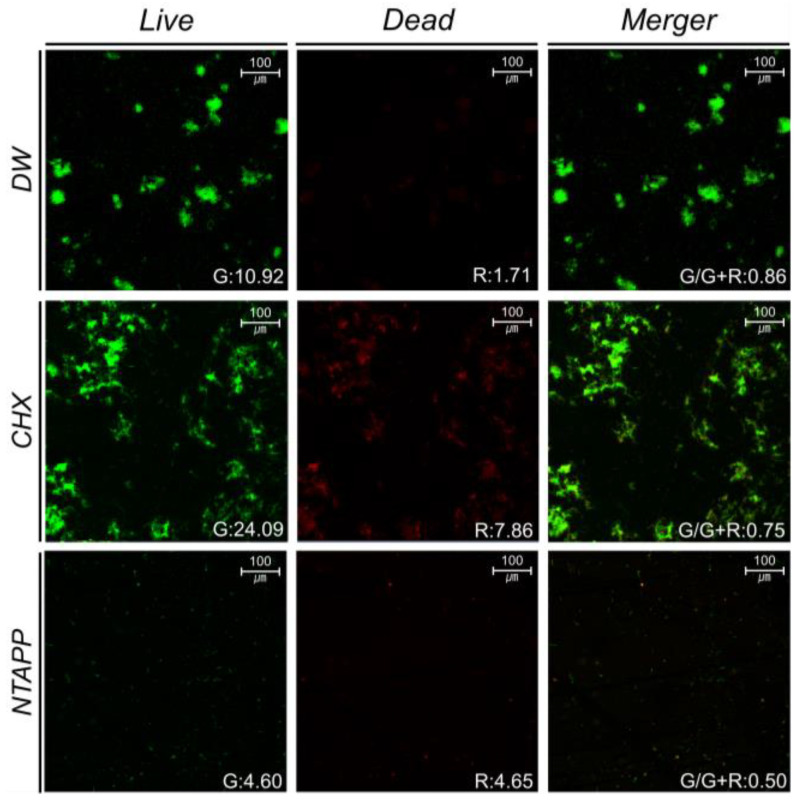
Representative confocal laser scanning micrographs (×100) showing the live (green-stained) and dead (red-stained) bacteria adherent to HA disks (CHX, chlorhexidine; DW, distilled water; HA, hydroxyapatite; NTAPP, non-thermal atmospheric pressure plasma).

The amount of viable bacteria (stained with a green fluorescent stain) was lowest in the NTAPP group. The amount of dead bacteria (stained with a red fluorescent stain) was lowest in the DW group. Bacterial viability (Ratio_G/G+R_) was 0.39 ± 0.09 lower in the NTAPP group (0.53 ± 0.04) than in the DW group (0.92 ± 0.08) and 0.22 ± 0.04 lower in the NTAPP group than in the CHX group (0.75 ± 0.00). In particular, the results indicated that bacterial auto-aggregation was inhibited in the NTAPP group (Figure 7).

## 4. Discussion

The antimicrobial effect of NTAPP in oral microcosm biofilms was evaluated to explore its potential as an alternative to CHX. Based on red fluorescence intensity (Ratio_R/G_), the NTAPP group had the lowest Ratio_R/G_ at all treatment durations (Figure 4) and Ratio_R/G_ gradually decreased as time went by. Our findings indicated that NTAPP had greater antimicrobial activity than CHX. Biofilm structures become more complex and thicker over time, resulting in increased resistance to stress and antimicrobial agents [26]. Nevertheless, NTAPP seems to have acted more efficiently than CHX in reducing the pathogenicity of mature biofilms. Although the 6-d-old biofilms with pathogenicity were exposed to NTAPP, ROS and RNS generated during plasma application likely contributed to the destruction of the bacterial membrane and suppressed the growth of the biofilm. Previous studies have reported that plasma treatment can have an antimicrobial effect [27,28]. After treating the biofilms with nitrogen-based NTAPP for 2 or 10 min, the excretion of cytoplasm in *Streptococcus mutans* was examined via transmission electron microscopy images, and the authors argued that the finding was clear evidence of cell injury [27]. Furthermore, Yoo et al. [28] found using scanning electron microscopy that pre-treating *S. mutans* biofilms with nitrogen-based NTAPP led to a lower number of adhered bacteria. These findings support the significant antimicrobial effect of NTAPP observed in this study.

Regarding the total number of live bacterial colonies and bacterial viability, total bacterial and aciduric bacterial counts were substantially reduced after NTAPP treatment compared to the DW treatment, confirming the antimicrobial effect of NTAPP. However, the NTAPP and CHX groups showed similar total bacterial counts, while the aciduric bacterial count was substantially lower in the CHX group (Figure 6). Nevertheless, bacterial viability (Ratio_G/G+R_) was the lowest in the NTAPP group and bacterial aggregation was inhibited in the NTAPP group compared to other groups (Figure 7). This suggests that NTAPP treatment can destroy the interaction site as well as the bacterial membrane itself [29]. A previous study reported that exposing *S. mutans*, *Streptococcus aureus*, *Klebsiella oxytoca*, and *Klebsiella pneumoniae* to NTAPP led to a substantially lower biofilm formation rate compared to that of the control group [27]. Further, they analyzed the viability of these four species of bacteria and found that the number of viable bacteria substantially decreased after NTAPP treatment compared to the control treatment [27]. Other studies have suggested that ROS of plasma can disturb bacterial aggregation by oxidizing extracellular polysaccharides (EPS) [30,31]. These previous results support our findings that NTAPP treatment could provide the lowest bacterial viability. Although the aciduric bacterial count was higher in the NTAPP than in the CHX group, biofilms with low bacterial viability may have lower pathogenicity compared to biofilms treated with CHX.

NTAPP was effective in reducing the pathogenicity, bacterial counts, and bacterial viability in oral microcosm biofilms. In this study, NTAPP was used as a small, hand-held jet device, such as a pipette, to irradiate sites with compressed plasma via a 1-mm nozzle. Small plasma equipment increases the accessibility of oral cavities and can thus be used to treat localized areas with clinical signs. Furthermore, when ions and neutral particles are produced in NTAPP, electrons are at a high temperature only in a short mean free path and thus do not induce thermal injury at the treatment site [10]. Thus, NTAPP treatment may be effective in controlling biofilms accumulated in areas that cannot be readily accessed by a toothbrush, such as deep periodontal pockets, pits, and fissures on the occlusal and proximal tooth surfaces. Therefore, NTAPP treatment is a potential candidate technology to replace chemical antimicrobial agents with several side effects.

This study had a few limitations. First, the cytotoxicity of harmful gases potentially produced by NTAPP treatment in the oral cavity could not be tested. In particular, concentrations of ozone and nitrogen dioxides generated from the NTAPP device are regulatory issues in medical equipment. A previous study reported that the cell viability of mouse fibroblast L-929 treated with an NTAPP jet for up to 2 min did not differ from that of the untreated group [28]. Another study reported that the reactive species created in plasma are inherently non-toxic because they are short-lived [20]. However, the cut-off concentration of ROS or RNS for determining whether a concentration is toxic to the cell or promotes cell metabolism may still be controversial [9]. Therefore, future studies should support the clinical utilization of NTAPP by testing relevant cytotoxicity and safety. Second, the distance from the nozzle during plasma irradiation may affect the NTAPP temperature and thus may be a risk factor for soft tissue [32]. However, we consistently maintained the distance between the biofilm and the nozzle of the Plasma Pipette^®^ at 10 mm. Therefore, further research will be needed to explore the appropriate irradiation distance that prevents damage to teeth and periodontal tissues. Third, we set the treatment time to 5 min considering the diameter (approximately 1 mm) of the plasma stream irradiated onto a 7-mm disk and the output pressure of 0.02 MPa. However, a 5- min treatment time could be too long in clinical settings. Thus, in a future study, the treatment time should be shortened by adjusting the length of the pipette nozzle and adding oxygen to the working gas. Finally, oral microcosm biofilms were formed on HA disks, as opposed to human enamel. This may influence the early attachment of the biofilm. However, we prepared the surfaces of HA disks using the same protocol for preparing specimens with human enamel [33]. Therefore, we minimized the potential effects of the HA disk surface on biofilm formation.

Despite the limitations of this study, our results suggest that NTAPP treatment may be effective in reducing the pathogenicity of oral microcosm biofilms. Furthermore, a strength of this study is that it substantiated the antimicrobial effects of NTAPP using an oral microcosm biofilm that closely resembles the actual oral environment. Based on the results of this study, it could be utilized as an alternative to CHX by maximizing the strengths of plasma.

## 5. Conclusions

Our findings suggest that NTAPP treatment was effective in reducing the pathogenicity and number of bacteria and weakening aggregation of oral microcosm biofilms. Therefore, NTAPP treatment can be used as an alternative to existing chemical antimicrobial agents.

## Data Availability

The data presented in this study are available from the corresponding author upon request.
